# Supplementary material for: Abnormal expression of GABAA receptor subunits and hypomotility upon loss of gabra1 in zebrafish
Source: Biol Open. 2020 Apr 28;9(4):bio051367. doi: 10.1242/bio.051367 (PMC7197724; doi:10.1242/bio.051367)
Supplement: Supplementary information [file biolopen-9-051367-s1.pdf]

Supplemental Figure 1

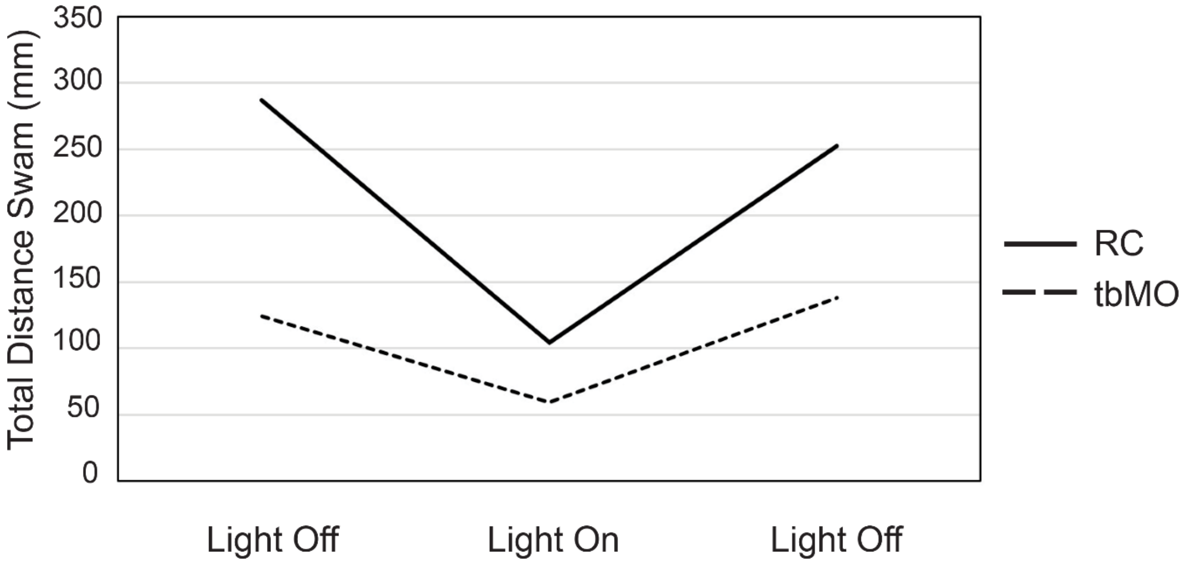

Figure S1: Hypomotility in *gabra1* morphants in alternating dark-light conditions. Total distance of larvae injected with random control morpholinos (RC) or translational targeting *gabra1* morpholinos (tbMO) was determined using Zebrabox technology at 5 days post fertilization (DPF). Distance was calculated without light, after the onset of light for a 5 minute duration, and an additional period without light.

Supplemental Figure 2

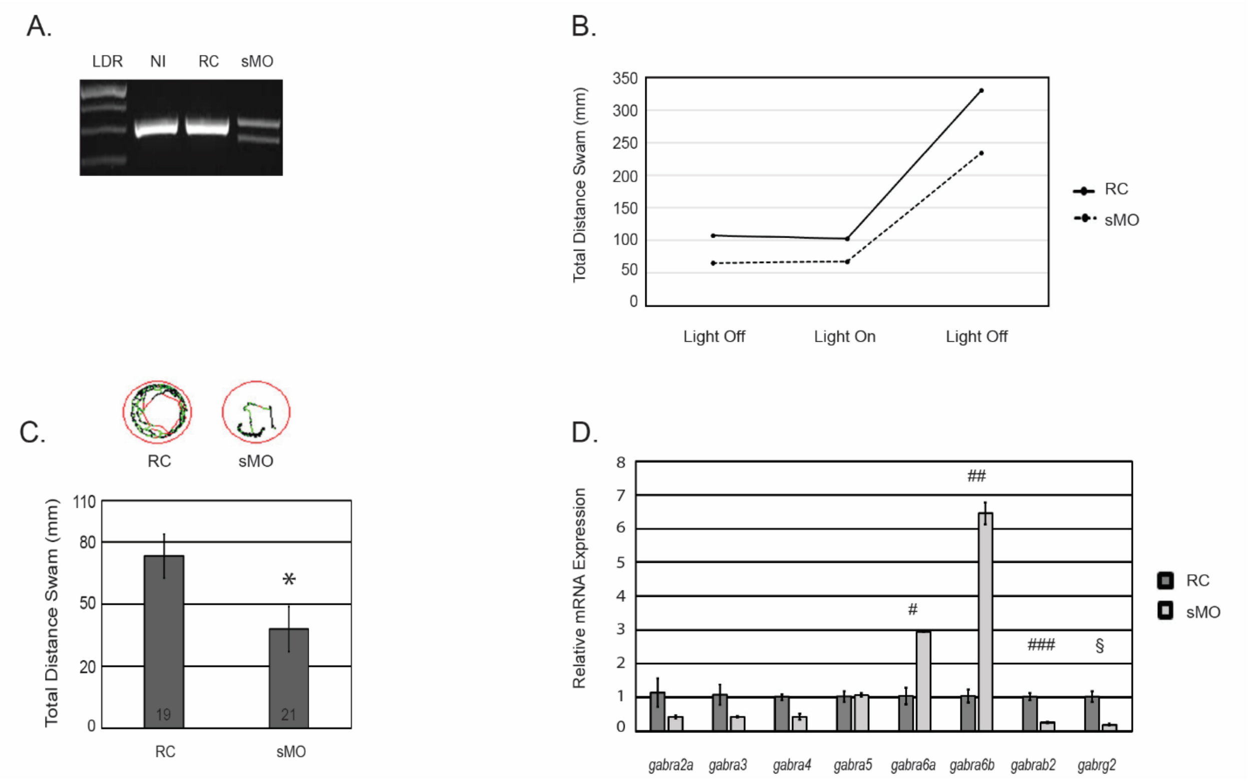

Figure S2: Knockdown of *gabra1* induces defects in splicing, hypomotility, and gene expression changes. (A) PCR analysis of alternative splicing was performed on non-injected control embryos, embryos injected with random control morpholinos (RC), or morpholinos inhibiting *gabra1* mRNA splicing (sMO). LDR is molecular weight ladder. (B) The total distance swam was assessed in *gabra1* morphants (sMO) and random control (RC) injected larvae at 5 days post fertilization (DPF). Distance was calculated without light, after the onset of light for a 5 minute duration, and an additional period without light. (C) The total distance swam was assessed at 5 DPF using Zebrabox technology. *$p<0.0263$. Representative images of larval swim patterns are depicted above graph. (D) Quantitative real time PCR (QPCR) was performed at 5 DPF to measure the expression of each gene indicated. Total RNA was isolated from random control injected embryos (RC) or *gabra1* targeting morpholinos (sMO). Error bars represent standard deviation. Expression was measured in biological triplicate. $^{\#}p=0.0087$, $^{\#\#}p=0.0001$, $^{\#\#\#}p=0.003681$, $^{\S}p=0.9024$

Biology Open • Supplementary information

TABLE S1. SUMMARY OF NEXT GENERATION SEQUENCING STATISTICS

	Proband	Mother	Father
	**NGS statistics**		
Raw reads	168,649,368	159,679,186	78,385,962
Reads mapped to hg19	165,308,012	156,265,642	76,698,260
Reads after duplicate removal	66,521,226	65,637,640	65,295,304
Mapped bases	6.65 Gb	6.56 Gb	6.53 Gb
Reads mapped to coding region	35,499,550	34,317,271	33,562,592
Average coverage of coding region	75.04 X	72.59 X	70.89 X

TABLE S2. SUMMARY OF EXOME VARIANTS AND TEST OF INHERITANCE MODELS

	**Proband**				
Total variants	106,737				
Coding variants	18,693				
Nonsynonymous, splice-site, InDel variants	9,631				
Rare variants	1,846				
Test of inheritance model		Dominant model	Recessive models		
		de novo	Compound heterozygous	Homozygous	X-linked hemizygous
	Candidate Genes	2 (*CACNA1C, GABRA1*)	5 (*SCNN1B, FNIP1, TTN, OTOG, FAT4*)	0	0
	Top Candidates	1 (*GABRA1*)	1 (*TTN*)	0	0

Biology Open • Supplementary information
